# 2248. Understanding attitudes and perceptions of younger adults in relation to antibiotic use for respiratory illnesses: Results from the Sore Throat and Antibiotic Resistance (STAR) study

**DOI:** 10.1093/ofid/ofad500.1870

**Published:** 2023-11-27

**Authors:** Sabiha Essack, Sergio Caretta-Barradas, Antonio Pignatari, Khalid Eljaaly, Wirat Tongrod, Adrian Shephard, Alike van der Velden

**Affiliations:** Antimicrobial Research Unit, College of Health Sciences, University of KwaZulu-Natal, Durban, KwaZulu-Natal, South Africa; Respiratory Disease Centre, Mexico City, Estado de México, Mexico; Paulista School of Medicine (EPM), Federal University of São Paulo (UNIFESP), São Paulo, Sao Paulo, Brazil; King Abdulaziz University, Saudi Arabia, Jeddah, Saudi Arabia; Faculty of Pharmaceutical Sciences, Huachiew Chalermprakiet University, Samut Prakan, Samut Prakan, Thailand; Reckitt Benckiser Healthcare International Ltd, Slough, England, United Kingdom; University Medical Center Utrecht, Utrecht, Utrecht, Netherlands

## Abstract

**Background:**

The World Health Organization is leading initiatives to drive public understanding of antibiotics and antimicrobial resistance. It is important to assess levels of understanding across countries and age groups.

**Methods:**

Observational, questionnaire-based study across 12 countries (Brazil, Germany, Spain, Italy, Mexico, Philippines, Poland, Romania, Saudi Arabia, Thailand, the UK, South Africa) explored respondent experience of respiratory illnesses and symptoms, antibiotic use, and attitudes to and perceptions of antibiotics. Respondents aged 18–64 years who had experienced and treated respiratory symptoms (selfcare and/or by a health care professional) in the past 6 months were included.

**Results:**

Of the 12,000 eligible adults (18–64 years) who completed the questionnaire, 6000 were from countries in Asia, Latin America, the Middle East, or Africa and 1816 were aged 25–34 years. Sixty-five percent of all respondents thought that they were quite knowledgeable about how antibiotics work on respiratory symptoms. However, answers to further questions indicated substantial misunderstanding. For example, several respondents believed that antibiotics kill viruses (65%), are effective for sore throat (73%), and relieve pain (65%). Typically, 25–34-year-olds had a lower understanding (68%, 74% and 67%, respectively). Almost half (48%) of all respondents felt they do not have enough knowledge on how to treat respiratory symptoms without antibiotics, which was similar in 25–34-year-olds (49%). Overall, 40% would feel anxious about being treated for respiratory symptoms without antibiotics; in 25–34-year-olds this response ranged from 33% (South Africa) to 55% (Thailand). There were misconceptions around when antibiotics should be stopped. For example, 40% of all respondents thought that you should stop taking antibiotics as quickly as possible; in 25–34-year-olds this response ranged from 29% (South Africa) to 52% (Philippines and Thailand).

**Conclusion:**

Although variable, there are still concerning misbeliefs surrounding antibiotic use for the management of respiratory illnesses, especially in the younger population. Different channels of communication may be required to increase health literacy on the appropriate use of antibiotics in the younger population.

**Disclosures:**

**Sabiha Essack, B. Pharm, M. Pharm, PhD**, Reckitt Benckiser Healthcare International Ltd, UK: Chairperson of the Global Respiratory Infection Partnership, supported by unrestricted educational grant from Reckitt Benckiser Healthcare Ltd|Reckitt Benckiser Healthcare International Ltd, UK: Member of the Global Hygiene Council, supported by unrestricted educational grant from Reckitt Benckiser Healthcare Ltd **Sergio Caretta-Barradas, MD**, Reckitt Benckiser Healthcare International Ltd, UK: Member of the Global Respiratory Infection Partnership, supported by unrestricted educational grant from Reckitt Benckiser Healthcare Ltd **Antonio Pignatari, MD**, Reckitt Benckiser Healthcare International Ltd, UK: Member of the Global Respiratory Infection Partnership, supported by unrestricted educational grant from Reckitt Benckiser Healthcare Ltd **Khalid Eljaaly, PharmD, MS, BCPS, BCIDP, FCCP**, Reckitt Benckiser Healthcare International Ltd, UK: Member of the Global Respiratory Infection Partnership, supported by unrestricted educational grant from Reckitt Benckiser Healthcare Ltd **Wirat Tongrod, PharmD, MBA, PhD**, Reckitt Benckiser Healthcare International Ltd, UK: Member of the Global Respiratory Infection Partnership, supported by unrestricted educational grant from Reckitt Benckiser Healthcare Ltd **Adrian shephard, BSc**, Reckitt Benckiser Healthcare International Ltd, UK: Employee **Alike van der Velden, PhD**, Reckitt Benckiser Healthcare International Ltd, UK: Member of the Global Respiratory Infection Partnership, supported by unrestricted educational grant from Reckitt Benckiser Healthcare Ltd

